# ARIAs are not random: A posterior and border‐zone vascular vulnerability model

**DOI:** 10.1002/alz.71304

**Published:** 2026-03-18

**Authors:** Alessandro Introna

**Affiliations:** ^1^ Department of Translational Biomedicine and Neuroscience, Neurology Unit University of Bari Aldo Moro Bari Italy; ^2^ Neurology Unit “L. Amaducci,” AOU Consorziale Policlinico Bari Italy

**Keywords:** amyloid‐related imaging abnormalities, border‐zone, posterior, posterior reversible encephalopathy syndrome, vulnerability model

## Abstract

- ARIA often shows posterior and watershed predominance, indicating region‐specific vascular vulnerability beyond amyloid burden alone.
- Reduced autoregulatory reserve in posterior territories may predispose to blood‐brain‐barrier disruption during anti‐amyloid therapy.
- Watershed regions are hemodynamically fragile, lowering the threshold for vasogenic edema or microhemorrhage after amyloid mobilization.
- ARIA may represent a PRES‐like neurovascular decompensation within an amyloid‐compromised vascular bed.

## 1 INTRODUCTION

The advent of anti‐amyloid therapy (AAT) has reshaped Alzheimer's disease (AD) therapy,^1^ but amyloid‐related imaging abnormalities (ARIAs) remain a key safety concern.^2^ ARIA‐E manifests as vasogenic edema and sulcal effusions, while ARIA‐H includes microhemorrhages and superficial siderosis, often interpreted as consequences of antibody‐mediated vascular amyloid clearance in cerebral amyloid angiopathy (CAA).^3^ Current models attribute ARIAs to transient blood–brain barrier (BBB) disruption and vessel wall instability after amyloid removal. Yet, the spatial distribution of ARIA lesions—frequently parieto‐occipital and involving watershed regions as reported by Zhang et al.^4^ —suggests a deeper, region‐specific vascular vulnerability.

It can be proposed that ARIAs preferentially affect posterior and border‐zone territories due to intrinsic susceptibility to autoregulatory failure and endothelial dysfunction. From this perspective, ARIAs may reflect regional vascular decompensation triggered by amyloid clearance rather than a uniform effect across the brain.

### 1.1 Regional vulnerability and mechanisms

Posterior circulation territories are particularly sensitive to autoregulatory failure, as exemplified in posterior reversible encephalopathy syndrome (PRES).^5^ Compared to anterior circulation, posterior vessels exhibit reduced sympathetic innervation, which diminishes dynamic autoregulation during abrupt blood pressure changes.^6^ This reduced buffering may predispose these regions to hyperperfusion and BBB disruption. In PRES, such dysregulation leads to vasogenic edema, predominantly parieto‐occipital. The radiological resemblance between ARIA‐E and PRES—both reversible vasogenic edema—suggests partially convergent mechanisms. While PRES is triggered by hypertension, cytotoxic agents, or systemic inflammation, ARIAs arise from pharmacologic endothelial stress superimposed on amyloid‐compromised vasculature.

In CAA, vascular smooth muscle degeneration, wall thickening, and endothelial impairment are already present. Antibody‐mediated amyloid mobilization transiently destabilizes this fragile balance. In posterior territories with limited autoregulatory reserve, this may suffice to precipitate vasogenic leakage.

Watershed regions—hemodynamic boundary zones between major arterial territories—are similarly vulnerable. Operating at lower perfusion pressures and with limited collateral reserve, they are predisposed to injury under systemic stress.^7^ Amyloid removal from leptomeningeal and cortical vessels during AAT may transiently alter vascular wall compliance and endothelial integrity, tipping these territories toward ARIA‐E or ARIA‐H.

### 1.2 Hemodynamic and amyloid contributions

If ARIAs were determined solely by vascular amyloid burden, a more uniform lobar distribution might be expected. Instead, recurrent posterior and watershed involvement suggests that vascular physiology contributes to lesion topography. Amyloid positron emission tomography studies in CAA demonstrate posterior‐predominant deposition, with higher occipital uptake than typical AD cohorts.^8^ Regional amyloid enrichment may amplify vulnerability in areas already predisposed to autoregulatory failure.

However, amyloid burden alone does not fully explain ARIA distribution. Hemodynamic factors also play a role. Post hoc analyses from anti‐amyloid trials identify elevated mean arterial pressure as a potential ARIA‐E predictor, while antihypertensive treatment reduces risk.^9^ Chronic hypertension impairs autoregulation and promotes microvascular damage. In a CAA‐compromised vascular bed, even modest blood pressure fluctuations may destabilize posterior and watershed regions, lowering the threshold for BBB disruption during amyloid mobilization.

### 1.3 Integrated vascular vulnerability model

These observations support a model in which ARIAs emerge from the interaction of regional amyloid deposition, endothelial fragility, and impaired autoregulatory reserve. AAT does not simply “cause” ARIAs; it unmasks latent instability within an already compromised cerebrovascular network. Traditional interpretations frame ARIAs as a direct toxic or inflammatory consequence of amyloid removal, but this does not explain territorial selectivity. Posterior and watershed territories may represent the weakest vascular components. Amyloid mobilization transiently increases permeability and mechanical stress. Where autoregulatory mechanisms remain robust, compensation occurs; where reserve is limited, vasogenic edema or microhemorrhage develops.

Reframing ARIAs as a PRES‐like process in an amyloid‐laden vascular bed offers conceptual clarity.^10^ Both share vasogenic edema, posterior predominance, reversibility, endothelial dysfunction, and autoregulatory imbalance. The primary difference lies in the trigger—systemic hemodynamic or toxic stress in PRES versus antibody‐mediated amyloid mobilization in ARIAs. Downstream cascades of BBB disruption may overlap substantially. This perspective shifts emphasis from an amyloid‐centric model toward an integrated neurovascular paradigm.

## 2 CONCLUSION

ARIA distribution is unlikely to be incidental. Posterior and border‐zone predilection suggests that regional vascular vulnerability is central to pathogenesis. Rather than viewing ARIAs as a uniform consequence of amyloid clearance, they should be conceptualized as a territorial vascular decompensation: antibody‐mediated amyloid mobilization precipitates autoregulatory failure in anatomically predisposed regions.

Recognizing ARIAs as a regionally expressed, PRES‐like vascular event within an amyloid‐compromised bed may refine mechanistic understanding and guide safer implementation of disease‐modifying therapies in AD.

## CONFLICT OF INTEREST STATEMENT

The author declares no conflicts of interest. Any author disclosures are available in the Supporting Information.

## FUNDING INFORMATION

The author declares that no funding was received to support the research, authorship, or publication of this article.

## Supporting information




Supporting Information
